# The Laryngoscope in Theory and Practice

**Published:** 1912-06-22

**Authors:** 


### THE LARYNGOSCOPE IN THEORY AND PRACTICE.

### Some Hints on its Manipulation.

It is one thing to be able to use a laryngoscope, but quite another to be thoroughly expert in its manipulation, and any patient who has had the larynx inspected by two or three examiners can very readily appreciate the difference.

A good illumination is very necessary, in order to get a clear view of the minute detail required in the diagnosis of a difficult case. An electric head-lamp is convenient for examining patients in their own homes, and a very portable pattern can now be obtained with a metallic-filament lamp actuated by a small dry cell; but the pencil of light does not coincide with the line of vision, and it is therefore better in the consulting-room to use a forehead-mirror and a powerful light. The light should be placed a little behind the patient's head, so as not to illuminate his face, as this distracts the attention, and must be on the examiner's right hand when the mirror is worn, as is usual, over the right eye. The ready adjustment of the forehead-mirror is a little difficult, and the beginner will find it no waste of time to practise this in the absence of the patient; it must be so arranged that the eye, looking through the central aperture, sees the illuminated area in the centre of its field of vision. An error in this adjustment is a common cause of failure to see the larynx, and of tired eyes and headache after the attempt. It is usual to introduce the laryngoscope mirror in the right hand, but, as this hand is ordinarily used for forceps, etc., it is advisable to become accustomed to hold the mirror in the left hand for all examinations.

The patient is now directed to open the mouth and protrude the tongue, which is then held by the examiner's other hand. The manner of holding the tongue makes a considerable difference to the patient's comfort; it must not be pulled forcibly or it may be cut against the lower incisor teeth, and if these be sharp, they should be covered by a small piece of lint or linen; it should be held directly forward and not downward, for the latter position tends to make the dorsum arch upward. A very good plan is to hold the tongue between the thumb above and the middle finger below, leaving the index finger free to place against the upper incisor teeth; it can be used to retract the upper lip and moustache, and serves the very important purpose of keeping the examiner thoroughly in touch with the patient, so that he may steady or move the patient's head, and, if the latter make a sudden movement, the tongue is not hurt. This "keeping in touch" is an important point in all such examinations, and on the same principle the writer always uses a short tongue-depressor in examination of the fauces and places the last two fingers below the patient's chin and, in examination of the nose, presses them against the forehead. The patient is directed to breathe naturally through the mouth and the laryngoscope mirror is introduced; it is passed in a curved direction over the dorsum linguæ until it is lost to view or "sets" behind it; it is then raised upward and backward until its surface becomes fully visible. The soft palate is in this way elevated by the back of the mirror, which is pressed firmly against the pharyngeal wall; care is taken not to touch the back of the tongue, but the mirror must be laid against the wall of the pharynx if a good view is to be obtained; firm pressure without tickling will not cause retching. The shank of the mirror may be brought into the angle of the mouth and steadied there in order to avoid titillation of the fauces. Before its introduction the mirror is warmed over a spirit lamp until the mist which gathers on its surface has cleared off; the glass side should be held towards the flame so as not to overheat the back, and the temperature must always be tested against the examiner's hand before it is introduced; a burnt patient dreads the examination. If preferred, the mirror may be heated in hot water and quickly dried, or its face may be smeared with a thin layer of soap.

The first difficulty encountered is irritability of the throat; in most cases a firm and steady touch will obviate this. The patient should be instructed to breathe evenly in and out. Cocaine in 5 or 10 per cent. solution may be used, and should be applied chiefly to the base of the tongue, where the retching reflex appears to originate. Sipping cold water or sucking ice is sometimes useful, or the patient may suck a heroin or orthoform lozenge. In some patients, especially in alcoholics and dyspeptics, the hyperæsthesia is so marked that the mere protrusion of the tongue produces retching before the mirror is introduced, and in the worst cases a satisfactory examination is impossible until after a week's course of saline aperients, light diet, abstention from alcohol and tobacco, and the use of a simple nasal lotion or spray.

The other common difficulty is that the epiglottis conceals the interior of the larynx. This is often due merely to faulty adjustment of the mirror; the patient's tongue must be well protruded, the head thrown a little back and the mirror placed against the pharyngeal wall and not merely on the soft palate, for since the upper aperture of the larynx looks backward as well as upward, the mirror must be brought somewhat behind the larynx. In some larynges, however, the epiglottis is unduly dependent and hides the anterior part of the glottis. The patient should be directed to phonate on the sound "eh" rather than "ah"; the epiglottis is raised on singing a high note or an ascending scale, or, if the patient be directed to make a prolonged phonation, the larynx can often be well seen during the involuntary gasp which follows. In the most difficult cases, if a complete inspection is essential, the epiglottis must be raised by a curved retractor placed in the glosso-epiglottic fossa after thorough cocainisation. If a very voluminous tongue hides the view of the mirror when in position, the tongue must be very gently depressed with a spatula while the patient holds it out with his own hand.

